# Insufficiency of Bone Scintigraphy in Vertebral Lesions of Langerhans Cell Histiocytosis Compared to F-18 Fluorodeoxyglucose Positron Emission Tomography/Computed Tomography and Diagnostic Computed Tomography

**DOI:** 10.4274/mirt.58066

**Published:** 2015-02-15

**Authors:** Zehra Pınar Koç, Selçuk Şimşek, Saadet Akarsu, Tansel Ansal Balcı, Mehmet Ruhi Onur, Ferat Kepenek

**Affiliations:** 1 Fırat University Faculty of Medicine, Department of Nuclear Medicine, Elazığ, Turkey; 2 Fırat University Faculty of Medicine, Training and Research Hospital, Clinic of Nuclear Medicine, Elazığ, Turkey; 3 Fırat University Faculty of Medicine, Department of Pediatric Hematology, Elazığ, Turkey; 4 Fırat University Faculty of Medicine, Department of Radiology, Elazığ, Turkey

**Keywords:** Histiocytosis, Langerhans-cell, Bone, scintigraphy, FDG PET/CT

## Abstract

Langerhans cell histiocytosis (LCH) is a benign disorder related to the histiocytes which can infiltrate bone tissue. The most effective method for demonstrating severity of this disease is PET/CT and bone scintigraphy might show bone lesions. We present a seventeen year old male patient with disseminated LCH presented with exophtalmos and having multiple vertebral lesions which were identified by F-18 FDG PET/CT scan and diagnostic CT but not in the bone scintigraphy.

## Abstract (tr)

Langerhans hücreli histiositoz (LHH) histiyositlerle ilişkili kemiği infiltre edebilen iyi huylu bir hastalıktır. Bu hastalığın yaygınlığını gösteren en etkili metod PET/BT’dir ve kemik sintigrafisi de kemik lezyonlarını gösterir. Yaygın LHH tanısı ve egzoftalmus ile takip edilen, çoklu vertebral lezyonları F-18 FDG PET/BT ve tanısal BT tetkikleri ile saptanan ancak kemik sintigrafisinde tespit edilemeyen, on yedi yaşında erkek hastayı sunmaktayız.

## INTRODUCTION

Langerhans cell histiocytosis is commonly associated with dissemination of the disease predominantly in the bone and other tissues like lymph nodes, lungs, liver and central nervous system (1). Although CT and magnetic resonance imaging (MRI) are effective methods in the identification of the bone lesions located at skull, vertebra and pelvis (2), F-18 FDG PET/CT might show all the dissemination of disease as a whole body imaging method and provides more clear demonstration of soft tissue involvement like in lymph node, lung or spleen (3). Additionally MRI is particularly helpful for not only characterizing the lesions but also delineation of the local and systemic extent of the disease and follow-up of the patient (4). Our case report presents a patient with disseminated disease with multiple vertebral lesions shown by FDG PET/CT and diagnostic CT which were not observed in the bone scintigraphy.

## CASE REPORT

Seventeen year old boy with mass lesion in the right neck and weight loss attended to the hospital. He had a family history of malignancy in his sister. He had bilateral exophtalmos, cervical lymphadenopathy approximately 3 cm in diameter, icterus and ulcers in the scalp in physical examination.

**Computed Tomography**

The computed tomography images were obtained by a 64 channel multidetector CT device (Aquilion 64, Toshiba Medical Systems, Japan) with intravenous contrast medium administration and with slice thickness of 0.5 mm. The images were reconstructed in axial, coronal and sagittal planes and interpreted in soft tissue and bone windows. 

**Figure 1:**
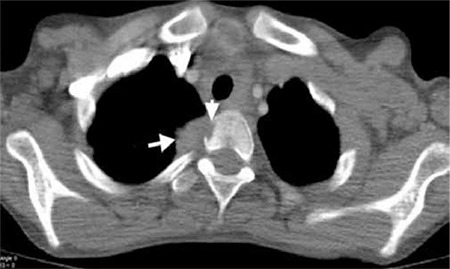
Axial CT images at bone window level demonstrate low attenuated right paravertebral soft tissue mass (arrows). Soft tissue mass invades body of the T2 vertebra and forms a low attenuated lesion in the vertebral body (arrowheads)

Computed tomography examination revealed multiple cervical, anterior mediastinal conglomerated lymph nodes and bilateral pulmonary nodules. Additionally lytic lesions in the corpus of T7, T10 and destruction of T2 vertebra by a soft tissue mass were reported in the diagnostic CT (Figure 1).

**Bone Scintigraphy**

The bone scintigraphy was performed by a double head single photon emission tomography (SPECT) gamma camera (GE, Infinia II, Israel) equipped with low energy high resolution parallel hole collimator. After intravenous administration of 20 mCi (740 MBq) (according to the body weight) Tc-99m methylene diphosphonate (MDP) and waiting period of 2-3 hours, routine whole body, and additional spot images were obtained. Asymmetrical increased tracer accumulation in unexpected sites without history of previous trauma or surgery was accepted as disease extent.

**Figure 2:**
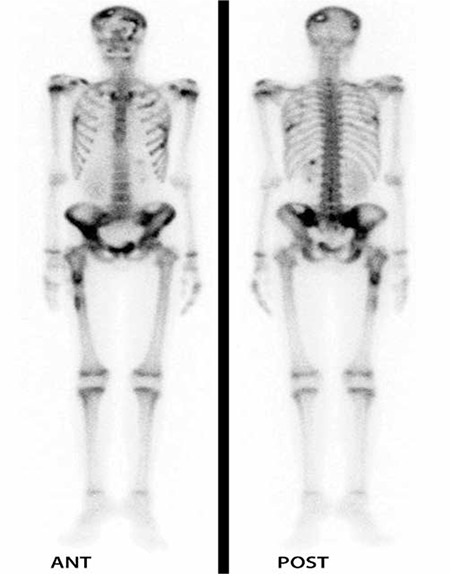
Anteroposterior whole body Tc-99m methylenediphosphonate bone scintigraphy shows disseminated disease with involvement of cranium, ribs and pelvic bones however no activity change is observed in vertebral column

Bone scintigraphy showed multiple increased activity in predominantly cranium, bilateral ribs and pelvic region (Figure 2) however, there was no sign of vertebral involvement; neither decreased nor increased activity accumulation.

**PET/CT Imaging**

The patient fasted for 12 hours before PET/CT examination and his blood glucose concentration was 124 mg/dL. Imaging was performed by a PET/CT scanner (Siemens Biograph, 6 slice spiral CT integrated PET/CT, Siemens Medical System) after injection of 10 mCi (370 MBq) 18F-FDG and 60 minutes waiting interval. CT was performed from head to thigh with a slice thickness of 5.0 mm. For semi-quantitative assessment, regions of interests (ROIs) were produced. The maximum SUV (SUVmax) value was measured from ROI according to the standard formula. PET and CT datasets and fusion images were interpreted by an experienced nuclear medicine physician.

In the PET/CT images there were multiple hypermetabolic areas bilaterally in cervical region, mediastinal, abdominal and pelvic lymph nodes, anterior mediastinal mass (Figure 3a) and in all the bone lesions identified in bone scintigraphy and CT (Figure 3b, 3c). Additionally orbital involvement of the LCH is observed as increased FDG accumulation in the left orbita and the soft tissue component in CT (Figure 3d, 3e). Excisional biopsy of cervical lymph node revealed Langerhans cell histiocytosis. In the biopsy specimen there were atypical histiocytes infiltrating the whole lymph node, giant cells and microabcesses were present. Immunohistochemical analysis revealed positivity of CD3, CD8, CD5, CD20, CD15, CD68, S-100, Vimentin, LCA and CD1a and negativity of CD10, Pan CK, EMA, Fascin, CD38 and CD138.

**Literature Review and Discussion**

Langerhans cell histiocytosis might cause death especially in the infants with pulmonary involvement (5). Bone is the most frequently affected site in 90% of patients which is the only site in one-third of the patients (6). Especially in childhood the bone lesions (50-57%) is the most frequent manifestation of the disease however in adult age the frequency varies (7). The incidence of vertebral involvement in LCH has been documented to be 12-35%, however a single lesion in the spine is relatively rare (8). LCH is characterized with lytic bone involvement which might cause predisposition to fractures in especially vertebral column. PET/CT shows lytic bone lesions more clearly than other types thus the diagnostic efficiency of PET/CT is superior to the other imaging methods in LCH. Aoki et al. have demonstrated that LCH lesions could have FDG uptake as high as osteosarcoma lesions (9). Coincidence FDG PET even has higher specificity than bone scintigraphy and radiography (10). Additionally PET/CT might show the treatment response of the LCH patients according to the previous studies (11). The presented patient had a critical lesion beneath the T2 vertebra which was identified in PET/CT. PET/CT identified a hypermetabolic lesion at second thoracic vertebra which had an apparent destruction on diagnostic CT in the presented patient. Although the lesion was more than 15 mm in diameter, the bone scintigraphy, secondary to a possible lytic nature of the lesion, was negative for this vertebral lesion. In the absence of a PET/CT the disease could be underestimated and treated inadequately.

Although Philips et al. have reported that bone scintigraphy identified approximately 75% of vertebral lesions in their series of 44 cases (3) bone scintigraphy was insufficient to show lytic vertebral involvement of LCH in our case. There is need for future prospective studies related to the diagnostic efficiency of bone scintigraphy in LCH. A previous study comparing FDG-PET to radiography and bone scintigraphy also have concluded that PET imaging is helpful in skull, limb, pelvis, scapula and clavicle lesions, however, not sensitive in spine which is attributed to complete collapse of vertebra or smaller diameter of lesions as shown by MRI (3). We in fact did not observe an underestimation of vertebral involvement of LCH in PET/CT, however, we did not observe the paravertebral effect of lesion to the adjacent structures to the T2 vertebra. This conclusion supports the idea of Azouz et al. that the lesions of LCH adjacent to the vital structures should be additionally evaluated by diagnostic CT (12). MRI has been also introduced as a suitable method for delineating the bone marrow extent and soft tissue involvement in LCH of the bone. Additionally MRI have found be superior in not only locating more skeletal lesions but also extraskeletal lesions (4,13).

Langerhans Cell Histiocytosis accounts for less than 1% of orbital tumors (14,15). In our patient one of the striking physical examination finding and one of the first finding of the disease was left sided exophtalmos which was a consequence of a mass lesion approximately 4 cm in maximum diameter, invading the globe with intracranial projection and infiltrating the bone tissue. This orbital lesion was also FDG avid and demonstrated increased osteoblastic activity in the bone scintigraphy.

Pulmonary LCH is also a rare entity which is presented as interstitial lung disease. It is approximately 5% of interstitial lung disease however isolated lung disease in LCH is rare (16). The presented patient also had lung involvement with multiple lung nodules without FDG uptake with diameter of ≤1 cm.

We presented a case of LCH with exophtalmos and disseminated disease in especially bone tissues. Comparison of bone scintigraphy and F-18 FDG PET/CT scan findings in our patient revealed that the sensitivity of the bone scintigraphy was inferior to F-18 FDG PET/CT scan in vertebral lesions.

## Figures and Tables

**Figure 3a:**
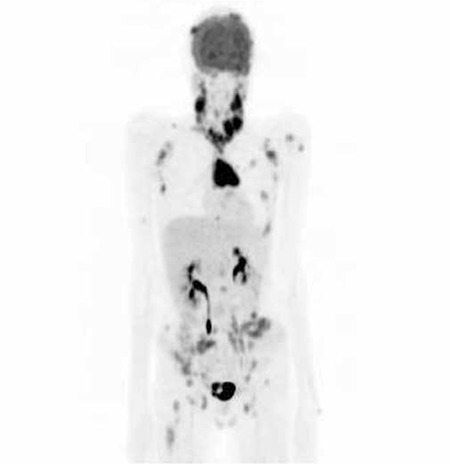
Multiple intensity projection image of F-18 Fluorodeoxyglucose positron emission tomography/computed tomography (FDG PET/CT) images of a patient with disseminated Langerhans Cell Histiocytosis. FDG accumulation of bilateral cervical, mediastinal, abdominal, pelvic lymph nodes and multiple bone lesions are present

**Figure 3b:**
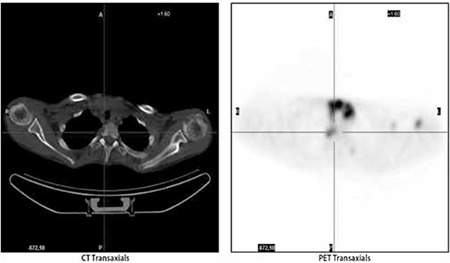
Axial CT part of the PET/CT study showing soft tissue involvement adjacent to the T2 vertebra

**Figure 3c:**
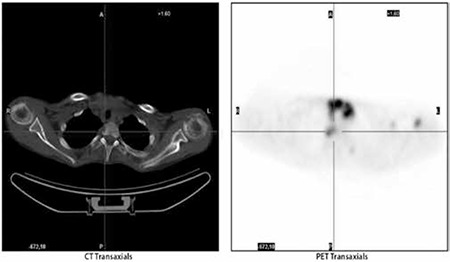
Axial PET part of the PET/CT study showing the FDG accumulation corresponding to the soft tissue involvement in the right side of the T2 vertebral body

**Figure 3d, 3e:**
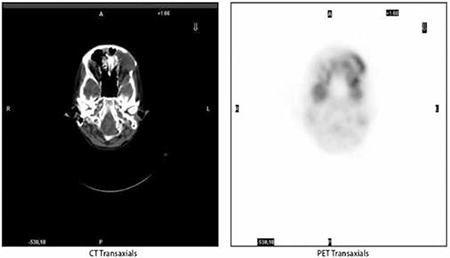
Left orbital involvement of the disease in axial CT and FDG accumulation corresponding to the soft tissue involvement in axial PET image respectively
